# The vascular endothelium masks the persistent inhibition of rat thoracic arterial tone induced by S-nitrosoglutathione

**DOI:** 10.5830/CVJA-2010-008

**Published:** 2011-02

**Authors:** M Sarr, AS Diallo, FB Sar, L Gueye, MO Kane, V Schini-Kerth, B Muller, A Wele

**Affiliations:** Laboratoire de Physiologie Pharmaceutique, Faculté de Médecine et de Pharmacie, Dakar, Sénégal; Unité Mixte Internationale (UMI – Environnement, Santé et Sociétés), Université Cheikh Anta Diop, Dakar, Sénégal; Laboratoire de Physiologie Pharmaceutique, Faculté de Médecine et de Pharmacie, Dakar, Sénégal; Laboratoire de Physiologie Pharmaceutique, Faculté de Médecine et de Pharmacie, Dakar, Sénégal; Unité Mixte Internationale (UMI – Environnement, Santé et Sociétés), Université Cheikh Anta Diop, Dakar, Sénégal; Laboratoire de Physiologie Pharmaceutique, Faculté de Médecine et de Pharmacie, Dakar, Sénégal; Unité Mixte Internationale (UMI – Environnement, Santé et Sociétés), Université Cheikh Anta Diop, Dakar, Sénégal; Laboratoire de Physiologie Pharmaceutique, Faculté de Médecine et de Pharmacie, Dakar, Sénégal; CNRS UMR 7213, Biophotonique et Pharmacologie, Illkirch, France; Pharmacologie, INSERM, Université Victor Segalen, Bordeaux, France; Laboratorie de Chimie therapeutique, Faculté de Médecine et Pharmacie, Dakar, Sénègal

**Keywords:** endothelium, nitric oxide, S-nitrosoglutathione

## Abstract

**Aim:**

In endothelium-denuded arteries, the nitric oxide (NO) donor S-nitrosoglutathione (GSNO) induced a persistent hypo-reactivity to vasoconstrictors, and low-molecular weight thiols such as N-acetyl cysteine (NAC) produced a relaxant effect. These effects were attributed to the formation of vascular NO stores. In arteries with a functional endothelium, such long-lasting effects on arterial tone have not been well characterised. In this study, we proposed to examine the possibility of storing exogenous NO when the vascular endothelium is still able to produce its own NO.

**Methods:**

For this purpose, changes in isometric tension of isolated arteries were assessed in organ chambers, and nitrosothiol formation was characterised by confocal microscopy.

**Results:**

In rat aortic rings with endothelium pre-exposed to GSNO, the contractile response to norepinephrine (NE) was not attenuated in comparison with control rings, but NAC induced a relaxant effect. However, an attenuation of the response to NE was observed in GSNO-exposed, intact aortic rings after inhibition of NO synthase by N^w^-nitro-L-arginine methylester (L-NAME) or in GSNO-denuded rings.

The relaxing effects of NAC were due to the mobilisation of NO from nitrosothiols after nitrosylation of protein SH residues. Moreover, the hypo-reactivity to NE and the relaxant effect of NAC were abolished by 1H-[1,2,4] oxadiazolo(4,3-a)quinoxalin-1-one (ODQ), an inhibitor of soluble guanylyl cyclase, and partially by the K^+^-sensitive channel inhibitor tetra-ethyl-ammonium (TEA).

**Conclusion:**

These data show that endothelium-derived NO masked the persistent effect of GSNO in rat thoracic aorta. However, the ability of GSNO to form releasable NO stores without altering the vascular tone can be particularly useful in preventing endothelial dysfunction in which NO formation decreases.

## Summary

Numerous in vitro and *in vivo* studies have demonstrated that in vascular diseases, the ability of the endothelium to secrete NO is reduced.^1^-^8^ Therefore, endothelium-independent nitric oxide donors might be useful to prevent or reverse endothelial dysfunction. Moreover, nitrosothiol (RSNO) formation from biotransformation of NO donors can take part in the transnitrosation reaction, which is a tranfer of bound NO from one thiol group to another, that under appropriate conditions, can release NO.^9^ NO donors such as nitrosoglutathione (GSNO) have been developed as valuable tools for experimental pharmacological studies and probably will be used in the future to restore vascular protection in pathological blood vessels,^10^-^12^ or to prevent vascular dysfunction.

Furthermore, little data exist on nitrosylation of thiols in healthy vascular tissue, and even less on functional consequences of this phenomenon on vasomotor activity. Therefore, the influence of endothelium on mechanisms through which nitric oxide donors can contribute to the hypo-reactivity of contractile agonists in healthy vessels is not well elucidated. This study was an attempt to investigate the effect of GSNO in normal vessels and to functionally characterise the underlying mechanism whereby this nitric oxide donor enhanced arterial hypo-responsiveness and relaxation.

## Methods

Experiments were conducted in accordance with the *Guide for the Care and Use of Laboratory Animals* as adapted and promulgated by the US National Institutes of Health (agreement number B 67900, given by French authorities). The thoracic aorta was removed from male Wistar rats (12–14 weeks old, 300–380 g) after anaesthesia with pentobarbital (60 mg/kg, i.p.) and cleaned of connective tissue and fat in Krebs solution (composition in mM: NaCl 119; KCl 4.7; MgSO_4_ 1.17; CaCl_2_ 1.25; KH_2_PO_4_ 1.18; NaHCO_3_ 25; glucose 11). The endothelium was removed by rubbing the intimal surface of the rings with forceps.

Changes in isometric tension of isolated arteries were assessed in organ chambers. The rings were allowed to equilibrate for 60 min before experiments were carried out, while the resting tension was adjusted, as required. Rings from various types of arteries were first exposed to GSNO (1 μM) or solvent for 30 min. After a 60-min washout period for drug removal, they were pre-contracted with norepinephrine (NE). Once the contraction reached a steady-state level, NAC was added. Parallel experiments were performed using N^w^-nitro-L-arginine methylester (L-NAME, an inhibitor of NO synthase), 1H-[1,2,4] oxadiazolo(4,3-a)quinoxalin-1-one (ODQ, a selective inhibitor of guanylyl cyclase), and tetraethylammonium (TEA, as a nonselective blocker of potassium channels).

For the characterisation of S-nitrosothiols, rat aortic smooth cells (RASMCs) were cultured in Labtek® chamber slides to confluence and then exposed to 100 μM S-nitrosoglutathion for 30 min. They were washed three times, then treated with HgCl_2_ (0.5 mM) or NAC (0.1 mM) and washed again. The cells were then fixed for one hour in 4% paraformaldehyde in PBS (0.1 M, pH 7.4) for one hour. They were then incubated for at least three hours at room temperature with a primary polyclonal antibody directed against S-nitrosothiols residues [1/100 diluted in a solution of PBS-Triton 0.5% (v/w)], followed by a secondary anti-rabbit IgG antibody coupled with fluorescein (Alexa Fluor® 488) diluted 1/200 in PBS-Triton. The preparations were then observed by confocal microscopy (Bio-Rad 1024 MRC®) with an epifluorescence at 40 × magnification.

To confirm and quantify the formation of nitrosylated protein, rat thoracic aorta (with and without endothelium) were first exposed to 100 μM GSNO for one hour at 37°C in organ chambers, followed by several washouts. The aortic rings were transferred into 24-well tissue-culture plates containing 0.2 ml of Krebs solution. The content of S-nitrosothiols (S-NO) was then measured using the Saville-Griess assay, involving diazotation of sulfanilamide and subsequent coupling with N-(1-naphtyl)ethylenediamine, after selective displacement of the NO group from the nitrosothiols by Hg^2+^ (0.5 mM). During the whole experimental period, the samples were protected from light.

Unless otherwise indicated, drugs were purchased from Sigma Chemical Co or Aldrich (Saint Quentin-Fallavier, France). Rabbit polyclonal antibodies directed against conjugated NO-cysteine were obtained as previously described.^13^ Alexa Fluor 488 was purchased from Molecular Probes (Leiden, the Netherlands). Horseradish peroxidase-labelled antibody (goat anti-rabbit IgG) was purchased from Diagnostic Pasteur (Paris, France). NAC (Fluimucil) was a generous gift from Zambon Laboratory (Antibes, France). Sodium pentobarbital was purchased from Sanofi Santé Animale (Libourne, France). GSNO was prepared as previously described.^14^

## Statistical analysis

Values are expressed as means ± SEM. Statistical evaluation was performed with the Student’s *t*-test for paired data or ANOVA, followed by Fischer’s protected least-significant difference test where appropriate. Values of *p* < 0.05 were considered statistically significant.

## Results

## Effect of GSNO exposure on rat aorta

**Fig. 1.**
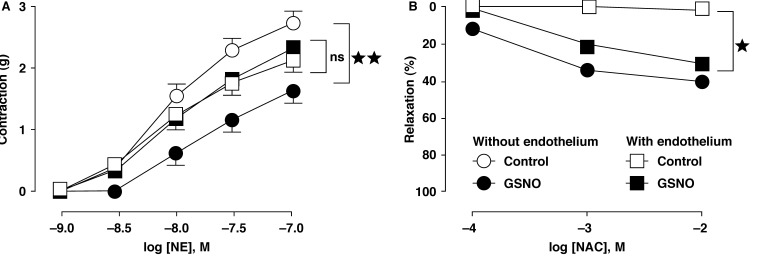
Effect of contractile and relaxant agonists in aortic rings pre-exposed to GSNO. (A) Pre-exposure to GSNO (1 μM, 30 min) was associated with a decrease in contractile response to norepinephrine, NE (1 nM to 30 μM) only in rat thoracic aortic rings without endothelium. (B) The low-molecular weight thiol NAC, which selectively cleaves NO bound from nitrosothiols (S-NO) after GSNO exposure, exerted a relaxant effect in rings both with and without endothelium. Results are means ± SEM of four to eight experiments; ns: not significant; *p < 0.05; **p < 0.01, in comparison with respective controls.

In endothelium-denuded rings from rat aorta pre-exposed to GSNO (1 μM, 30 min), the contractile response to NE (1 nM to 30 μM) was diminished in comparison to the controls (Fig. 1A). However, in rings with endothelium pre-exposed to GSNO, the effect of the contractile agonists was not affected (Fig. 1A). In both rings, NAC (0.1–10 mM) exerted a relaxant effect (Fig. 1B). The relaxing effects of NAC demonstrated the existence of releasable NO stores in the vessels with as well as those without endothelium. However, the vascular endothelium masked the existence of these stores because only the vessels without endothelium showed the hypo-responsiveness characteristic, indicating the presence of these stores.

**Fig. 2.**
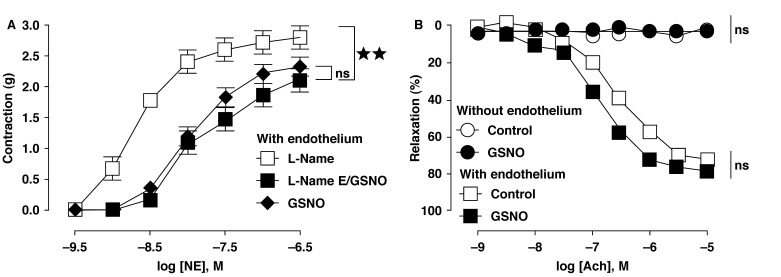
Blunted GSNO-induced hypo-responsiveness to contractile agonist in aortic rings with endothelium was associated with an endogenous NO production which only accounted for vasorelaxation to acetylcholine. (A) shows a decrease of vascular tone after GSNO exposure to rings with endothelium in the presence of the nitric oxide synthase (NOS) inhibitor, N-nitro-L-arginine methylester (L-NAME, 300 μM). (B) indicates that acetylcholine-induced relaxation was not affected by GSNO exposure in rings with and without endothelium and did not display vasorelaxation to acetylcholine even after GSNO exposure. Results are means ± SEM of four to six experiments; ns: not significant; **p < 0.01 in comparison with respective controls.

As expected, in rings with endothelium in the presence of the NO-synthase inhibitor L-NAME (300 μM), when added to NE 15 min before the contractile response, we observed the same decrease in vascular tone as seen in the rings without endothelium after GSNO addition (Fig. 2A). These data indicate that NO storage remains an effective mechanism of formation of NO stores after NOS blockade.

Our study also shows that the acetylcholine-induced relaxation was not affected by the addition of GSNO to rings with intact endothelium (Fig. 2B). By contrast, rings without endothelium did not display vasorelaxation to acetylcholine even after GSNO exposure, suggesting that only endothelium-derived NO and not those from exogenous sources account for vasorelaxation to acetylcholine.

## Formation and quantification of S-nitrosothiols

**Fig. 3.**
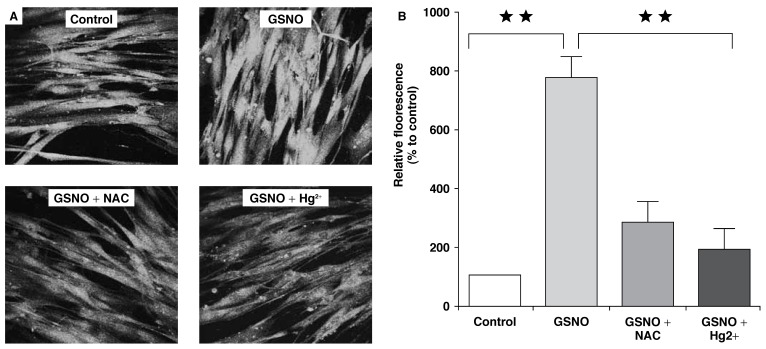
Nitrosothiol formation as a storage form of NO in the vasculature. (A) shows immunostaining of S-nitrosylated residues in rat aortic smooth muscle cells (control) and after in vitro exposure to GSNO (100 μM, 30 min) or after GSNO exposure and treatment with mercuric chloride (GNSO + Hg^2+^) or N-acetyl cysteine (GSNO + NAC). (B) indicates corresponding cumulative data shown as means ± SEM of three different experiments. **p < 0.01 in comparison with respective controls.

In order to confirm the ability to store exogenous NO in the vascular wall, we proceeded to characterise the S-nitrosylated residues following exposure of rat aortic smooth muscle cells to GSNO. As indicated in Fig. 3, S-nitrosylated residues were detected using polyclonal antibodies directed against the S-NO moiety. A weak staining was observed in the rings treated with mercuric chloride or NAC (which selectively cleaves NO bound from S-NO) after exposure to GSNO. This was similar to what was observed in the control, which was not exposed to GSNO. These data clearly indicate the presence of nitrosothiols as a storage form of NO in the vasculature.

**Fig. 4.**
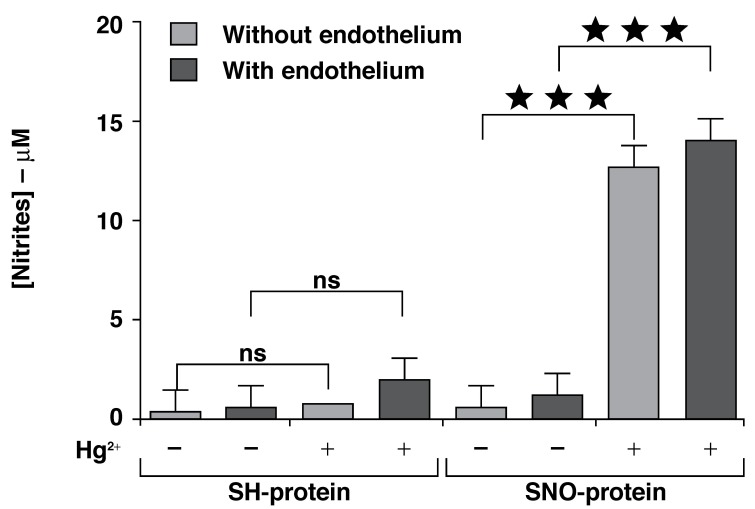
Quantification of nitrosothiols in rat thoracic aorta. Rat aortic rings were subjected to the S-nitrosylation assays and incubated with 100 μM GSNO for 30 min (SNO-proteins) or solvent (SH-proteins). Nitrite formation was determined with the Saville-Griess assay after selective displacement of the NO group from nitrosothiols with 0.5 mM mercuric chloride (Hg^2+^). Data are means ± SEM of three independent experiments.

However, quantification of nitrosothiols with the Saville-Griess reaction (Fig. 4) has shown that in aortic rings both with and without endothelium and not exposed to GSNO (SH-proteins), NO released by Hg^2+^ was not significantly elevated. By contrast, the NO mobilised by the same conditions and transformed to nitrite was significantly higher when the rings were exposed to GSNO (SNO-proteins). However, the amount of nitrite measured was not significantly different in the two rings after GSNO exposure, showing once again that the NO storage was the same in the rings with and without endothelium.

## Effect of ODQ in GSNO-treated aortic rings

**Fig. 5.**
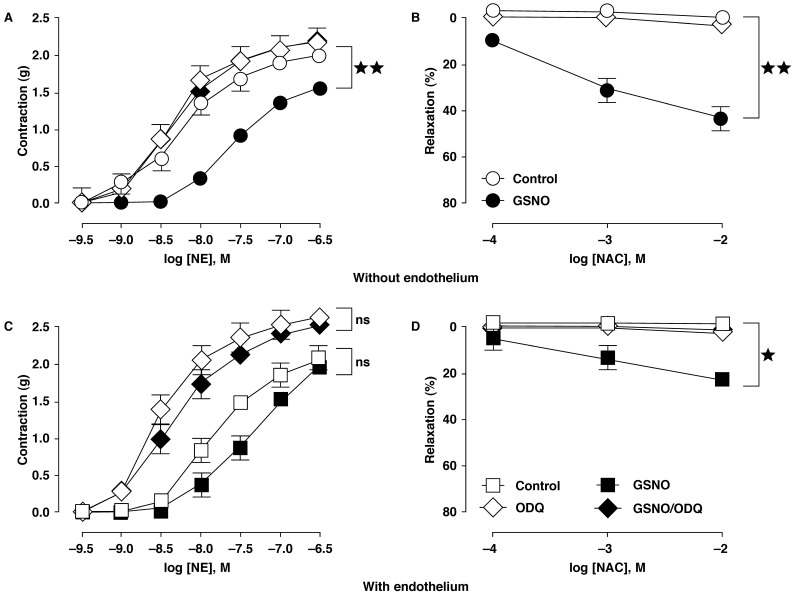
The GSNO-induced hypo-reactivity and associated relaxation mechanisms involved the NO/cGMP pathway. (A) shows that the guanylate cyclase inhibitor, ODQ (1 μM, 30 min) had no effect in denuded control rings, but completely abolished the loss of reactivity to NE in GSNO-treated denuded rings. By contrast, (C) indicates that ODQ increased the tension of both controls and GSNO-treated intact rings by reversing the NO-induced hypo-responsiveness to NE. (B) and (D) demonstrate that soluble guanylyl cyclase mediated the relaxant effect of NAC in the controls and GSNOtreated aortic rings with or without endothelium. Results are means ± SEM of five experiments; ns: not significant; *p < 0.05; **p < 0.01 in comparison with respective controls.

To determine the involvement of guanylyl cyclase in the hyporesponsiveness induced by GSNO and the relaxant effect of NAC, we studied the effect of ODQ. Aortic segments were exposed to 1-μM ODQ for 30 min and then stimulated with a cumulative concentration of NE. When the contractile response reached a plateau, cumulative concentration-effect curves for NAC were constructed. Our data show that ODQ (1 μM, 30 min) increased the tension of both control and GSNO-treated intact rings (Fig. 5A, Table 1) by reversing the NO-induced hypo-responsiveness to NE. By contrast, there was no effect in the denuded control rings, but complete abolishment of the loss of reactivity to NE in the GSNO-treated denuded rings (Fig. 5C, Table 1). NAC-evoked relaxations in GSNO-treated rings with and without endothelium were abolished by ODQ, which was similar to the controls (Fig. 5B, D). These findings indicate that the mechanisms accounting for the hypo-responsiveness to agonist-induced contractions and also the relaxing effect of NAC involved activation of the soluble guanylyl cyclase.

**Table 1.** Contractile Effect Of Norepinephrine Expressed In Grams Of Developed Tension (E-MAX) And EC_50_ Values, Determined By Log-Logit Regression In Aortas Pre-Exposed To Gsno And Inhibitors

	*Control*	*GSNO*	*ODQ*	*GSNO/ODQ*	*TEA*	*GSNO/TEA*
Without endothelium						
E-max (g)	2.46 ± 0.082	1.93 ± 0.074*	2.74 ± 0.16^ns^	2.66 ± 0.045^ns^	2.12 ± 0.162^ns^	1.76 ± 0.123*
EC_50_ (nM)	5.31 ± 1.392	30.55 ± 4.963***	3.74 ± 0.915*	4.30 ± 1.464^ns^	0.59 ± 0.063**	6.77 ± 0.154^ns^
With endothelium						
E-max (g)	2.04 ± 0.184	2.07 ± 0.123^ns^	2.62 ± 0.178^ns^	2.51 ± 0.094^ns^	2.42 ± 0.093^ns^	2.39 ± 0.179^ns^
EC_50_ (nM)	15.07 ± 1.077	46.64 ± 2.035**	2.48 ± 0.086**	4.04 ± 0.041**	5.20 ± 0.144**	14.10 ± 0.131^ns^

Data are means ± SEM of five experiments; ns: not significant; *p < 0.05; **p < 0.01 ***p < 0.001 in comparison with respective controls.

## Inhibition of the TEA-sensitive K^+^ channels

**Fig. 6.**
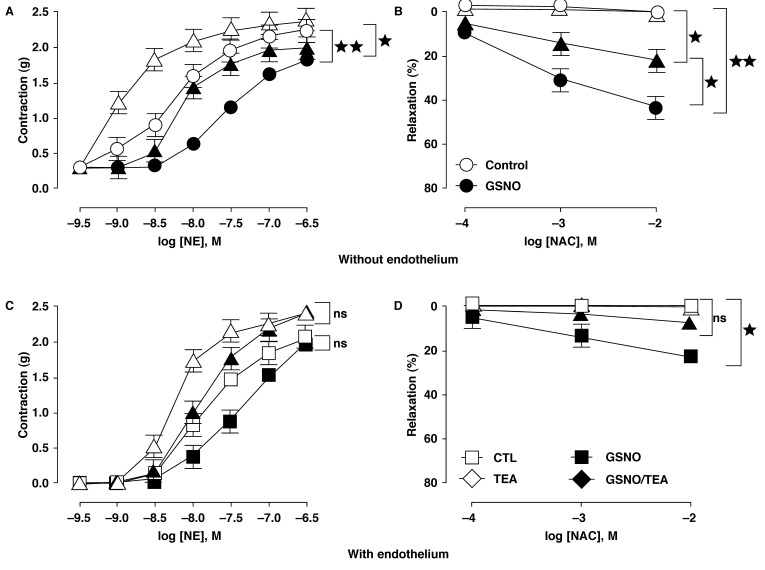
The GSNO-induced hypo-reactivity and associated relaxation mechanisms partially involved the activation of TEA-sensitive K^+^ channels. (A) and (C) established that the unselective K^+^ channel blocker TEA (10 mM, 30 min) caused a large increase in sensitivity to NE of both controls (not exposed) and endothelium-denuded or intact rings pre-exposed to GSNO. (B) and (D) indicate that the effects of NAC were partially inhibited by TEA on GSNO-treated denuded or intact rings. Results are means ± SEM of five experiments; ns: not significant; *p < 0.05; **p < 0.01 in comparison to respective controls.

Aorta segments pre-exposed to GSNO were incubated with K^+^ channel blockers 30 min before the administration of the cumulative dose of norepinephrine. When the contractile response reached a plateau, cumulative concentration-effect curves for NAC were created. Our results show that TEA (10 mM, 30 min) had no affect on the GSNO-induced loss in vessel reactivity but increased the sensitivity of control and GSNO-exposed rings without endothelium (Fig. 6A, Table 1). Similar findings were observed with control and GSNO-exposed rings with intact endothelium (Fig. 6C, Table 1). The effects of NAC were partially inhibited in GSNO-treated rings pre-incubated with TEA (Fig. 6B and 6D).

These findings also indicate that the mechanisms accounting for the hypo-responsiveness to agonist-induced contractions and the relaxing effect of NAC partially involved the activation of TEA-sensitive K^+^ channels.

## Discussion

This study provides new insights into the functional interaction between endothelium-derived NO and endothelium-independent nitric oxide donors. Our data demonstrated that in rings with intact endothelium from rat aorta pre-exposed to GSNO, there was no change in vascular reactivity following the administration of the contractile agonist norepinephrine, but the low molecular weight thiol, NAC, exerted a relaxant effect. These results provide evidence that NO stores can be formed in the presence of intact endothelium. These data confirm and complete previous studies from the existing literature in which it was reported that among NO donors, some of them (S-nitrosating agents) induced a long-lasting vasorelaxation, especially in endothelium-denuded arteries.^9^,^15^-^17^

One of the interesting results reported in this present study is the hypo-responsiveness observed in vessels treated with the NO synthase inhibitor L-NAME after GSNO exposure. Indeed, these results suggest that in vessels with endothelial dysfunction in which a significant decrease in the production of NO is observed, it is possible to maintain the vascular tone at normal levels. Moreover, our results showed that treatment with GSNO did not alter the relaxations induced by endogenous vasorelaxation agents such as acetylcholine.

Our results also indicate that the inhibitor of cGMP production in smooth muscle, ODQ, enhanced norepinephrine contraction in control rings with intact endothelium and GSNO-treated rings from rat aorta, but had no effect in the denuded control rings. ODQ also abolished NAC-evoked relaxations in GSNO-treated rings with and without endothelium, similar to that seen in the controls. Since the attenuation of the contractile response induced by GSNO and the relaxation effects of NAC were abolished by ODQ, this hypo-reactivity and the associated relaxation mechanisms involved the NO/cGMP pathway. These results confirm previous studies that soluble guanylyl cyclase mediates vasorelaxant activity associated with endogenous NO production or exogenous sources such as nitric oxide donors.^15^,^18^-^20^

Other observations support the idea that ODQ is less potent in inhibiting relaxations by NO, therefore implicating a component of NO-induced relaxation that is independent of sGC/cGMP.^21^ The important endogenous production of NO could therefore mask the effects of an additional supplementation with nitric oxide donors under these conditions. Several data indicated that in rat aorta the NO-synthase inhibitor, L-NAME, completely abolished the relaxation to acetylcholine,^22^,^23^ suggesting that in these arteries, the activation of the NO/cGMP pathway appears to be more relevant than the relaxing pathway.

It may also suggest that GSNO could desensitise the sGC, as mentioned in the work of Sayed *et al.*^24^,^25^ Indeed, exposure to nitric oxide and to S-nitrosothiols caused S-nitrosylation of sGC, which directly desensitised sGC to stimulation by nitric oxide. They also reported that S-nitrosylation and desensitisation were prevented by treatment with N-acetyl-cysteine (NAC), a precursor of glutathione, clinically used to prevent development of nitrate tolerance.^24^ In another article, the same authors reasoned that sGC, as the main or even the sole receptor activated by NO, could be targeted by S-nitrosylation to induce its desensitisation, thus constituting an exquisite process of sGC modulation by negative feedback.^25^

They tested the hypothesis that sGC desensitisation was induced by S-nitrosylation and showed that (i) the NO-stimulated activity of semipurified sGC was reduced by pre-treatment with GSNO and correlated with its S-nitrosylation; (ii) NO-stimulated activity was reduced by ~ 50% with 50 μM GSNO pre-treatment, compared with GSH.^25^

Our results also indicate that the unselective K^+^ channel blocker TEA caused a large increase in sensitivity of the rings to NE without modifying the maximal response to the agonist in rings denuded of endothelium. In a previous work, Terluk *et al.* had already reported that the non-specific potassium channel blockers TEA and charybdotoxin, a Ca^2+^-activated K^+^ channel blocker, inhibited the hypo-responsiveness to phenylephrine induced by the NO donors.^15^

By contrast, 4-aminopyridine, an inhibitor of voltage-gated potassium channels, and glibenclamide, which specifically blocks ATP-sensitive K^+^ channels, had no effect. Furthermore, opening of potassium channels, more specifically the calcium-activated subtype, plays a predominant role in this NO-induced hypo-responsiveness to phenylephrine in the rat aorta.^15^ Finally, it was also established that incubation of NO donors with rat aortic rings induced substantial reduction in phenylephrine-induced contractions. This effect was long lasting and involved calcium-dependent potassium channels.^26^

Some reports indicate that in small vessels such as the mesenteric arteries, the contribution of voltage-dependent and Ca^+^-activated large-conductance K^+^ channels appears to be more relevant than that of the NO/cGMP pathway.^27^,^28^ Other data also indicate that NO and its donors can directly stimulate BK(Ca) activity in cells isolated from the rat mesenteric artery. This ability of NO to directly open BK(Ca) channels could play an important functional role in NO-induced relaxation of the vascular smooth muscle cells in this low-resistance artery.^29^ S-nitrosothiols can also activate SK(Ca) + IK(Ca) channels. Since S-nitrosothiols decompose to NO, stored S-nitrosothiols may mediate bradykinin-induced, EDHF-dependent relaxation.^30^

Moreover, soluble guanylate cyclase activation and opening of potassium channels play an important role in NO-induced hypo-responsiveness to norepinephrine in the rat aorta.

## Conclusion

The present study demonstrated that formation of releasable NO stores remained effective in rings with a functional endothelium, but endothelium-derived NO blunted the hypo-responsiveness to GSNO-induced vasorelaxation. Not only can GSNO simulate NO-induced hypo-responsiveness to contractile agonists without modifying the basal tone in isolated, intact vessel, but this model could also be particularly useful to prevent endothelial dysfunction when vascular failure could be imminent.
